# Accelerating the Delivery of Psychological Therapies After Stroke: A Feasibility Stepped-Wedge Cluster Randomised Controlled Trial

**DOI:** 10.3390/healthcare13070824

**Published:** 2025-04-04

**Authors:** C. Elizabeth Lightbody, Kulsum Patel, Emma-Joy Holland, Chris J. Sutton, Christopher Brown, Svetlana V. Tishkovskaya, Audrey Bowen, Jessica Read, Shirley Thomas, Temitayo Roberts, Caroline L. Watkins

**Affiliations:** 1 School of Nursing and Midwifery, University of Central Lancashire, Preston PR1 2HE, UK; kpatel@uclan.ac.uk (K.P.); clwatkins@uclan.ac.uk (C.L.W.); 2 Population Health Sciences Institute, Faculty of Medical Sciences, Newcastle University, Newcastle upon Tyne NE2 4AX, UK; emma.holland@ncl.ac.uk; 3 Lancashire Clinical Trials Unit, University of Central Lancashire, Preston PR1 2HE, UK; cjsutton3@uclan.ac.uk (C.J.S.); cbrown24@uclan.ac.uk (C.B.); stishkovskaya@uclan.ac.uk (S.V.T.); 4 Division of Psychology and Mental Health, School of Health Sciences, University of Manchester, Manchester M13 9PL, UK; 5 East Lancashire Hospitals NHS Trust, Pendle Community Hospital, Leeds Rd., Nelson BB9 9SZ, UK; jessica.read@elht.nhs.uk; 6 Centre for Rehabilitation and Ageing Research, School of Medicine, University of Nottingham, Nottingham NG7 2UH, UK; shirley.thomas@nottingham.ac.uk; 7 NHS Cheshire and Merseyside Integrated Care Board, 1 Lakeside, 920 Centre Park, Warrington WA1 1QY, UK; temitayo.roberts@cheshireandmerseyside.nhs.uk

**Keywords:** stroke, psychological support, mood disorders, feasibility studies, stepped-wedge design

## Abstract

**Background:** Psychological problems post-stroke are common and debilitating, yet insufficient evidence-based psychological support exists for stroke survivors, either in stroke or general mental health services. Many stroke survivors with significant needs remain unsupported. To address this problem, we need pathways to identify, treat and manage psychological difficulties after stroke. The Accelerating Delivery of Psychological Therapies after Stroke (ADOPTS) study aimed to explore the feasibility of collaboratively developing, implementing and evaluating intervention packages (IPs) to facilitate access to, and increase the provision of, psychological support post-stroke. **Methods:** Stakeholder groups were formed across four sites in north-west England, comprising stroke and psychological services, to collaboratively develop site-specific IPs incorporating a psychological care pathway, staff training, a staff manual for stroke-specific psychological support and supervision. A feasibility stepped-wedge cluster randomised trial recruited patients admitted with stroke during the usual care (pre-implementation of the IP) and intervention (post-implementation) periods. The feasibility of IP implementation and their potential usefulness were evaluated through assessing wellbeing and the support received, and through a process evaluation incorporating interviews with staff, patients and carers. Feasibility evaluation included the recruitment rate and attrition rate; exploratory analysis (mixed-effects linear or logistic regression models) was used to assess the ‘promise’ of the intervention in achieving psychological distress outcomes (mood (PHQ-9), anxiety (GAD-7)), assessed using validated measures at 6 weeks and 6 months. **Results:** IPs were collaboratively developed at each site but implementation took longer than the per-study-protocol duration of three months. Nineteen training sessions (152 attendees) were delivered for nursing, therapy, NHS Talking Therapies and voluntary staff. Nursing staff were underrepresented due to difficulties with releasing staff. Manuals were developed for each site, incorporating a mood screening and referral algorithm, but these were not finalised at one site. Stroke and NHS Talking Therapies champions were identified in each site to facilitate cross-service staff supervision. A total of 270 patients were recruited over 14 months (133 usual care, 137 intervention), with 227 and 198 at 6 weeks and 6 months, respectively. Stroke staff found the training, manual and pathway helpful, and reported greater confidence in managing and referring psychological issues. NHS Talking Therapies staff found the training useful for adapting their therapy. However, the intervention took longer to implement in all sites, requiring an additional time period to be added to the stepped-wedge design. **Conclusions:** It is feasible to collaboratively develop and implement IPs for post-stroke psychological support. However, an alternative to the stepped-wedge design used here would be more appropriate for a future study. This study was registered in ISRCTN—the UK’s Clinical Study Registry (trial registration: ISRCTN12868810, registration date: 4 February 2016).

## 1. Background

Stroke affects over 100,000 people in the UK each year [1]. Although more people than ever will survive, they may be left with disabilities [2], which in turn may affect their psychological wellbeing, with depression [3], anxiety [4], emotionalism [5] and post-traumatic stress disorder (PTSD) [6] being common. Psychological difficulties can significantly impact the individual and their recovery. Depression, affecting approximately one in three stroke survivors [3], is associated with poorer outcomes, including increased healthcare utilisation [7], poorer functional outcomes [8], reduced quality of life [9], higher rates of suicide [10] and mortality [11,12], in addition to higher costs [13].

Despite being highlighted by government bodies as an important issue for post-stroke care, psychological difficulties often go undetected [14] and psychological care remains largely unavailable to many stroke survivors [15]. A United Kingdom (UK) survey found that over one-fifth of stroke survivors felt that the emotional changes were difficult to deal with, with one-quarter waiting up to five months for psychological support [16]. One reason for this is the dearth of specialist psychology staff, with less than two-thirds of stroke units in the UK (*n* = 112, 61.2%) having access to clinical psychology [17]. These service gaps mean that stroke survivors, often with huge psychological needs, are left unsupported [16].

To address this problem, we need pathways to identify, treat and manage psychological difficulties after stroke. Stroke guidelines recommend a collaborative approach utilising a matched-care model [18,19] comprising three levels (or ‘Steps’) of care, in which people start at the most appropriate level of care for their needs. The model assumes that most patients will experience mild to moderate difficulties (Steps 1 and 2), and these patients can be best supported by stroke-specific staff. Specialist psychology staff, such as Clinical Psychologists/Neuropsychologists, provide higher level support (Step 3) to those with severe difficulties and supervise staff working at lower levels. Utilising a matched-care model allows for the best use of specialist staff’s limited time, whilst ensuring patients receive the correct level of support. Evidence suggests that a collaborative, matched-care approach, where the intensity of psychological intervention is tailored to individual patient needs, can effectively address psychological problems [20,21,22].

However, access to specialist psychology services post-stroke varies greatly both geographically and within NHS Trusts, for example, between acute and rehabilitation services. Additionally, despite the fact that people from areas of greater deprivation have an increased risk of stroke and are more likely to experience more severe strokes [23], health inequalities based on socio-economic status may limit access to psychological support. Such factors require examination to identify and understand the barriers and facilitators to accessing and receiving psychological support.

The inconsistency in psychological care may be addressed through increasing collaboration between stroke services and generic psychology services, such as NHS Talking Therapies (formerly known as Improving Access to Psychological Therapists; IAPT), to increase access to psychological support and specialist psychology staff. NHS Talking Therapies services use a matched-care approach, similar to the stroke-specific model; whilst the stroke matched-care model comprises three steps, the NHS Talking Therapies model has four steps. In NHS Talking Therapies, junior psychology staff treat problems at Steps 1 and 2, with more senior and specialised staff treating more severe problems at Steps 3 and 4. NHS Talking Therapies have been effective in reducing anxiety and depression in the general population and have been encouraged to widen access to those with long-term conditions [24,25]. However, although many NHS Talking Therapies services have long-term condition champions, the number of services which have worked with patients following stroke is unknown. Stroke survivors may be perceived as challenging due to stroke-related impairments, such as cognitive or communication difficulties, which may hinder receiving traditional talk-based psychological therapies. Training NHS Talking Therapies teams in stroke-specific issues might increase confidence in, and capacity for the delivery of, Step 2 and 3 care for stroke survivors. Conversely, stroke services often focus on physical health, and staff may lack the knowledge, skills or experience in managing psychological distress [26]. Training stroke staff to deliver Step 1 psychological support, and in doing so, increasing their confidence, would facilitate the application of the matched-care model.

A complex intervention involving increasing collaborative work between services and delivering staff training needs robust evaluation. Challenges to implementing this complex intervention need to be understood. Therefore, it was decided that a feasibility study should be conducted to capture implementation issues to be considered for a larger trial. A stepped-wedge cluster randomised controlled trial (RCT) design [27] was selected in which all clusters (sites) started in the control phase, and each site received the intervention at staggered points in the study timeline. The cluster design also mitigated the potential contamination risks involved in an individual patient RCT for this type of service-level intervention.

The aim of the ADOPTS study was to develop and implement an intervention package and to explore the feasibility of utilising a collaborative approach to delivering post-stroke psychological care within existing NHS, social care and voluntary sector services.

The study design encompassed several components, conducted in three phases:

Phase 1: Describe current pathways for psychological support post-stroke and the challenges of provision and access.

Phase 2: Based on Phase 1 findings, develop an evidence-based intervention package to facilitate access to, and increase the effectiveness of, health, social care and voluntary sector services focused on stroke survivors’ psychological needs.

Phase 3: Apply the intervention package and explore its feasibility, acceptability and potential effectiveness.

### Objectives

A: Evaluate the feasibility of collaboratively developing and implementing the intervention package.

B: Assess whether the development of the intervention package impacts psychological service provision prior to its implementation.

C: Estimate the eligibility, recruitment and attrition rates for a larger trial.

D: Develop and test data collection systems, outcome measures and follow-up protocols.

E: Estimate the proportion of people with psychological distress, the time to first referral and the time to treatment.

F: Explore the potential benefit of the intervention package for patients, including for different stroke types and socio-economic subgroups.

G: Investigate the feasibility of the stepped-wedge design to evaluate the delivery of the intervention package.

## 2. Methods

### 2.1. Study Design and Ethics

A feasibility stepped-wedge cluster randomised trial with embedded process evaluation. This study was registered in ISRCTN—the UK’s Clinical Study Registry (trial registration: ISRCTN12868810, registration date: 4 February 2016).

This study was reviewed by the NRES Committee Yorkshire and The Humber–Leeds East and received a favourable opinion (Rec reference 15/YH/0343).

### 2.2. Setting

Acute and community NHS Trusts based in four stroke services in the North of England.

### 2.3. Patient Carer and Public Involvement (PCPI)

A PCPI group was established for support throughout the study. Members included those with lived experience of stroke, or of caring for someone who had experienced a stroke. The group met regularly and were involved in the development of patient facing materials (information sheets, questionnaires) to ensure study materials were appropriately completed and accessible to stroke survivors and their carers, including those with communication difficulties.

### 2.4. Randomisation

**Figure 1:**
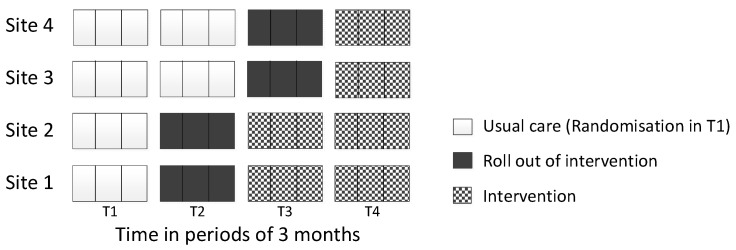
Stepped-wedge design.

The four stroke services (clusters) were randomised to one of two dates in pairs: two sites were randomised to start implementation at the first date and the remaining two sites were randomised to start implementation at the second date (3 months apart). Randomisation was performed by an independent statistician using computer-generated pseudo-random numbers. All clusters started in a usual care phase (no intervention delivered at any site, T1), then sequentially crossed over to the roll-out phase, which was intended to last 3 months, until all sites received the intervention (T4, see Figure 1).

### 2.5. Phase 1

Service mapping of current psychological services

To establish a picture of the psychological services across the study sites, we conducted a scoping and mapping exercise, including a patient record review, and held interviews with service users and staff. The results from the interviews will be reported in detail in future publications. To identify gaps in services and staff skills, information was sought regarding current psychological care pathways, including what psychological support was available, the mode of delivery, demands on current services and the identification of who accessed those services when they were available. This information was mapped to each site, which informed stakeholder group discussions regarding the development of intervention packages for each of the four sites during Phase 2.

Hospital Record Review

A retrospective review of patient hospital records was undertaken to explore if the process of the scoping and mapping exercise and the development of the implementation package may have had an impact on the psychological support patients received by raising staff awareness of unmet needs. Consecutive stroke patients on four acute stroke units were identified over two time points: (i) one week prior to process mapping and the development of the implementation package, and (ii) one week during the roll-out period of the implementation package. For those who consented, the hospital records were reviewed by a member of the research team who looked for instances where psychological care (e.g., psychologist, psychiatrist, mental health liaison) was recorded. Quality assurance was assessed by a second member of the research team who independently extracted data for a sample of the hospital records (4–6 for each site).

### 2.6. Phase 2: Feasibility Trial

Participants and setting

Consecutive stroke patients admitted to acute stroke units at each of the four sites were identified during the middle month of each 3-month period (usual care, roll-out and intervention) (see Figure 1). Eligible participants included those who had survived to day 3 post-stroke or those who were discharged prior to day 3; those living within the catchment area of an NHS Trust; and those aged ≥18 or over. Exclusion criteria included those who lacked capacity and had no friend/relative/carer to act as consultee. The carers of patients were also invited to participate. For participants who lacked capacity, a consultee provided assent.

Intervention

The intervention package involved providing a patient pathway for psychological support after stroke. This pathway was adopted as part of usual care; therefore, patients consented to data collection and follow-up only. While the delivery varied slightly across sites, the core aspects (referral pathway, training, manual and supervision) of the implementation strategies were consistent across all sites. The implementation package was planned to be rolled out over a 3-month period to embed the pathway in the services prior to the start of formal data collection for the intervention phase.

Data collection

Baseline data collection

Baseline data were recorded for all participants who had provided valid consent. Data were extracted from (i) patient records, including their age, sex, date of admission, stroke side and severity; (ii) face to face including communication (FAST [28]) and cognition (MoCA [29]); and (iii) from patient self-report questionnaires, including mood (PHQ-9 [30], (DISCs) [31], anxiety (GAD-7 [32]), Posttraumatic stress disorder (IES-6) [33], stroke recovery (Modified Rankin [34], Barthel (3 items) [35], Short Form Stroke Impact Scale (SF-SIS) [36], Work and Social Adjustment Scale (WSAS) [37]) quality of life (EQ5D3L [38]) and indicators of one’s social/economic context (Supplementary Table S1). Staff were also asked to complete proxy measures of patient mood (SADQ-10 [39] and Yale single item [40]) and anxiety (BOA [41]) based on their perception of how they thought the patient was feeling. All measures and questionnaires have previously been validated for use with the study population.

Outcome data collection

All participants received a postal questionnaire at 6 weeks and 6 months post-stroke. Patients were given the option of telephone or face-to-face completion. Carers who consented completed questionnaires about their observations of the patient’s mood (SADQ-10 and Yale single item) and anxiety (BOA) at 6 weeks and 6 months post-stroke. Details of study measures are in Supplementary Materials Tables S1 and S2.

Statistical analysis

The analyses were conducted and reported following both the CONSORT 2010 statement extension for the reporting of stepped-wedge cluster randomised trials [42] and for reporting randomised pilot and feasibility trials [43]. As the trial aimed to assess the feasibility of intervention delivery to inform the design of a main trial, the indicative outcomes were not powered for the statistical testing of effectiveness.

Feasibility outcome measures were summarised using descriptive statistics with proportions of eligibility, recruitment, attrition rates and percentages of missing data estimated for each outcome measure at 6 weeks and 6 months.

To address the different aspects of feasibility and to inform the decision about a fully powered trial, the following participant numbers were summarised by their frequencies for each intervention group:

- Number of patients suffering from psychological distress (anxiety or depression according to the Psychological Distress Algorithm (Appendix A)) and who received psychological support at each time point;
- Number of patients with anti-depressant use at each time point;
- Number of patients when psychological treatment was first received;
- Number of patients who required a letter to be sent to their GP to notify them of a potential issue concerning psychological distress;
- Number of patients with further stroke, TIA or other major health problems which required hospital admission (electronic health records were compared to participant reported problems at each follow-up using kappa statistics);
- Number of reminders sent to encourage participants to return the questionnaires.

Baseline and demographic characteristics were summarised descriptively by the study arm using the median (interquartile range [IQR]) or proportions, as appropriate. Frequency distributions were examined for the variables describing the number of participants deemed to have anxiety or depression at baseline, at 6 weeks and at 6 months.

To explore the potential benefit of the intervention packages for different levels of deprivation, the numbers of participants suffering from psychological distress at 6 weeks and 6 months by deprivation index were examined using frequency distributions. Deprivation was measured using the Index of Multiple Deprivation (IMD) as quintiles and treated as a categorical variable.

To inform the main trial, we performed modelling of the outcome and process variables using generalised linear mixed models, with a mixed-effects logistic regression model used to model binary outcome variables (the computed psychological distress due to anxiety, psychological distress due to depression and psychological distress due to anxiety or depression) and a mixed-effects linear model for the PHQ-9 and GAD-7 scores, with site as a random effect. In addition to the intervention and cluster, the models also included time period factors and baseline outcome measures. Interaction terms between the type of stroke and the intervention group, and the IMD quintiles and the intervention group were subsequently included in the model to evaluate potential subgroup intervention effects.

The completeness of the outcome data was assessed through the response rate for the outcome questionnaires at each time point and the completion rate of the individual items. We also examined questionnaires to see if individual items were repeatedly not completed to determine participant accessibility or acceptability. Responders for psychological distress were compared to non-responders at each time point regarding their baseline characteristics and demographics, using logistic regression analysis to identify individual factors associated with non-response.

Missing data for each outcome at each time point (including baseline) was summarised overall by intervention group and by stepped-wedge design (site-by-time period). Multiple imputation was not deemed appropriate given the exploratory nature of the effectiveness analysis.

To assess the potential contamination of the usual care phase (T1) due to service mapping, the proportion of patients identified as having psychological needs, as being assessed for mood problems, as having been referred for psychological assessment/support and having started on anti-depressants before and after the service mapping were summarised and compared using logistic regression, which was adjusted for site.

For the analysis, we assumed that patients recruited during the roll-out period of the stepped-wedge design received usual care. A sensitivity analysis was conducted where participants recruited during the implementation period were assumed to have received the intervention.

Effect estimates were presented as point estimates with 95% confidence intervals. Where appropriate, the significance level was set to 5%. The descriptive analysis was performed using SPSS v.24 [44] and modelling performed using Stata v.15 [45].

### 2.7. Phase 3: Process Evaluation Interviews

Patient Process Evaluation Interviews

Following the roll-out of the intervention package, a sample of patients recruited to the main implementation package were interviewed to explore the acceptability of the tailored psychological service.

Participants and setting

Stroke patients and carers were purposely selected (n~12 per site) to recruit a balanced sample across sex, age (younger/older), stroke severity (mild/moderate/severe), communication abilities and time since stroke. Patients and their carers who consented were telephoned by a member of the University of Central Lancashire research team. For all of those with communication difficulties who consented to being contacted, a carer was approached, and for all of those with cognitive deficits, a consultee was contacted. Written informed consent was obtained prior to the interview commencing. Interviews were held face-to-face in the participant’s home, or by telephone, depending on participant preference.

Data collection

The interviews were audio recorded. For participants with communication difficulties, interviews were video-recorded with the patient’s consent. Patients and their carers (interviewed separately) were asked to describe their experiences of psychological support since their stroke, including what worked well, or which areas they felt could be improved. For participants with communication difficulties, the Supported Conversation for Adults with Aphasia (SCA^TM^) [46] techniques were used in the design of the interview. The interviewers also used these techniques to adapt their communication methods to be tailored to individual patient needs.

Qualitative data analysis

Interviews were transcribed and analysed using thematic analysis, and interpretation was underpinned by the Theoretical Domains Framework (TDF [47]). The TDF grouped the constructs identified from theories relevant to the implementation of healthcare interventions into 14 domains. Themes were identified and key concepts developed through the interpretation of patterns and were mapped onto the domains of the TDF.

Staff Process Evaluation Interviews

Following the roll-out of the implementation package, staff were interviewed about their experiences to understand their feelings towards implementing the intervention package, including the training and supervision received, what they felt worked well and what they felt could be improved.

Participants and setting

The participants were staff working across the stroke pathway in hospitals, in the community and in generic mental health services within each of the sites, including nurses, doctors, occupational therapists, physiotherapists, allied health professionals and NHS Talking Therapies therapists, reflecting the range of staff involved in the care pathway at each site. Staff were invited by a member of the research team. Written informed consent was obtained from all staff willing to take part.

Data collection

Staff took part in a semi-structured interview based on the TDF. Interviews were held face-to-face or by telephone, or face-to-face as part of a focus group, depending on participant preference.

Qualitative data analysis

Interviews were transcribed and thematically analysed using TDF.

## 3. Results

### 3.1. Objective A: Evaluate the Feasibility of Collaboratively Developing and Implementing the Intervention Package

**Figure 2:**
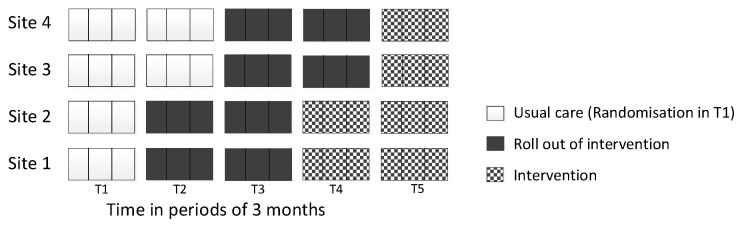
Modified stepped-wedge design implemented to include five phases.

Stakeholder meetings were held in all four sites to inform the development and implementation of the intervention package. There were 38 attendees (Site 1 = 10, Site 2 = 11, Site 3 = 8, Site 4 = 9), and in each site they included representation from stroke services (acute, rehabilitation, community), NHS Talking Therapies, Stroke Association and patients and carers. Using information gathered from stakeholder meetings and findings from the pre-implementation interviews, an intervention package was collaboratively developed, tailored for each site. The four core components (referral pathway, training, manual and supervision) of the IP were supplemented with additional resources following suggestions by staff and patients, including an information leaflet about psychological problems post-stroke, and a card displaying useful contacts for patients. The roll-out of the intervention took longer than the intended 3 months in all sites. Therefore, an additional time period was added to the stepped-wedge design to ensure all sites experienced a full intervention period (see Figure 2). Overall, each core component was implemented to varying degrees across sites.

#### 3.1.1. IP Component 1: Screening and Referral Pathway

A screening and referral pathway for psychological care was developed in all four sites. In one site, the pathway was developed but not fully implemented, as it required approval and authorisation at an executive level, which was not completed during the study period. In the other three sites, the pathway was implemented and in process evaluation interviews was reported to be useful when embedded as part of practice, with staff knowing how and when to refer issues and to whom.

#### 3.1.2. IP Component 2: Training

Training packages were developed and implemented in all sites, with training delivered separately to stroke and NHS Talking Therapies teams. Six planned training sessions were cancelled due to staff shortages; of these, three were rearranged and three did not take place because it was not possible for staff to be released. In total, nineteen training sessions were held across the four sites (Site 1: seven sessions, Site 2: two sessions, Site 3: three sessions, Site 4: two sessions; NHS Talking Therapies covering Sites 1 and 2: four sessions, NHS Talking Therapies covering Sites 3 and 4: one session). There were 152 attendees at the training sessions, including staff across various roles and levels of experience (42% therapy staff, 15% nursing staff (including HCAs), 32% NHS Talking Therapies staff and 11% Stroke Association staff). Of all staff in post across the four sites, 8% (*n* = 22/269) of nurses/HCAs and 59% of therapy staff (*n* = 64/108) attended the training sessions.

To facilitate attendance, training sessions were offered and held at different times (morning, afternoon, evening) and locations. At the suggestion of staff, training sessions were also aligned with existing team meetings. Despite this, some staff were unable to attend due to their workload, particularly nursing staff when wards were busy.

The degree of continued implementation of the training differed across sites. For example, in Site 2, training was cascaded to staff and was integrated into in-service training. In Site 4, however, the training for stroke teams was intended to be delivered by NHS Talking Therapies teams, but this was not possible during the study period due to time pressures, and so the stroke team only received written information from NHS Talking Therapies.

#### 3.1.3. IP Component 3: Manual

A manual was developed in all four sites. In one site, the manual required authorisation at an executive level before it could be used in practice, and this authorisation was not completed within the study period. In the other three sites, the manual was implemented by stroke teams in different ways. In one site, the manual was embedded as a fundamental resource for existing and new staff; in another site, the manual was available for staff who wished to use it. Across sites, some junior staff reported being unaware that the manual existed. Staff who were aware of the manual found it a useful resource for determining when and how to screen for mood problems.

#### 3.1.4. IP Component 4: Supervision

Supervision links were developed in all four sites, with a ‘stroke champion’ identified and contacts in stroke teams and NHS Talking Therapies teams named for reciprocal support. The names and contact details of these individuals were provided during training and within the manual; as such, there were differences between sites in the awareness and use of this information. Some staff reported that they had used the details of local stroke or NHS Talking Therapies champions to build links across teams. The supervision component was facilitated by stakeholder meetings and training, which allowed staff to meet and establish professional relationships and to provide assistance to each other. The contact details of stroke champions were also used in materials designed for patient use, e.g., key contact cards and information leaflets.

### 3.2. Objective B: Assess Whether the Development of the Intervention Package Impacted Psychological Service Provision

A review of patient hospital records was carried out in each site over two time points to check for potential contamination during the usual care phase due to the study set-up, service mapping and stakeholder meetings. The first hospital record review was undertaken in all sites one week prior to the study commencing, and the second held in a week during the study set-up phase. The hospital record review found no differences in how mood was routinely screened for or reported between pre-study and study set-up phases. Due to this, in the main analysis, participants recruited during the roll-out periods were assumed to have received usual care.

### 3.3. Objective C: Estimate the Eligibility, Recruitment and Attrition Rates for a Larger Trial

**Figure 3:**
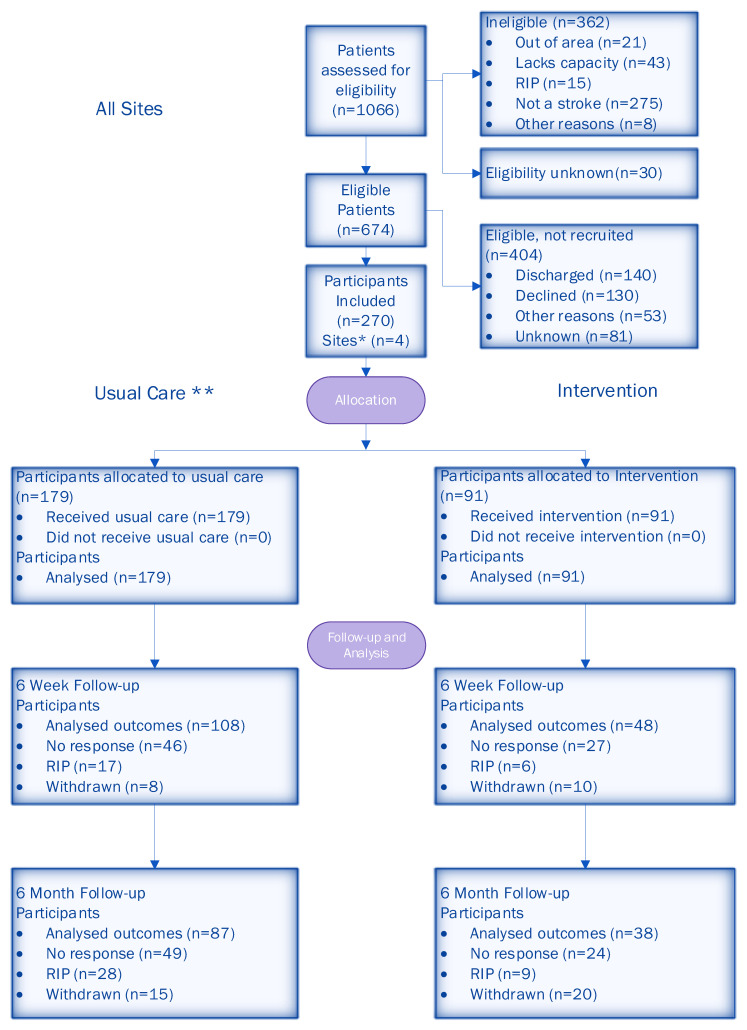
CONSORT diagram of participant flow through the trial. * All four sites provided data at each time point. ** Usual care included participants that were recruited during the roll-out period.

**Figure 4:**
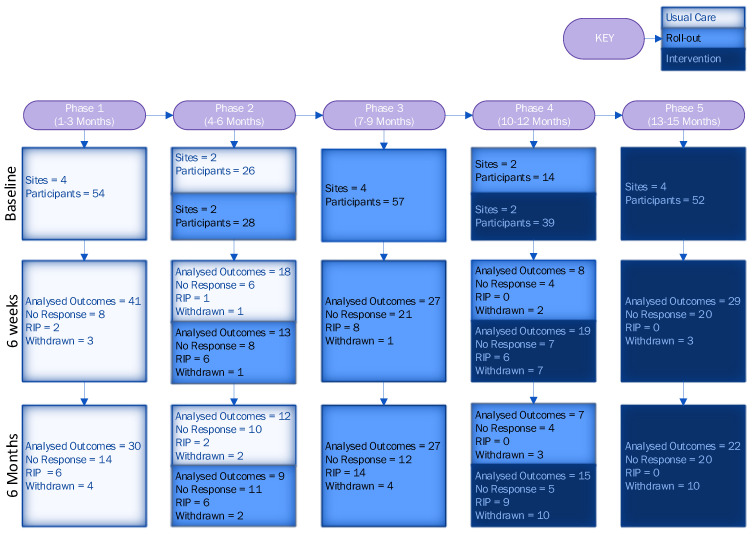
Flow chart of stepped-wedge design by allocated sequence, period and follow-up time.

A total of 1066 participants were screened for eligibility across four sites. Of those screened, 674 (63%) were deemed eligible. A total of 270 (40%) patients consented to participate, with 179 (66%) in the usual care period and 91 (34%) in the intervention period (the imbalance in allocation was due to patients in the roll-out period being treated as usual care). The CONSORT diagram (Figure 3) shows the flow of participants. Following the CONSORT extension for reporting stepped-wedge cluster RCTs [42], we also included a flow chart describing a stepped-wedge design by allocated sequence, period and follow-up time (Figure 4).

The baseline and demographic characteristics of participants by study arm are shown in Table 1.

The usual care and intervention groups were similar in terms of most characteristics. However, there were two notable differences, as shown in Table 1: (i) usual care participants were less likely to have ischaemic stroke, but more likely to have intracerebral haemorrhage; and (ii) usual care participants were more likely to use anti-depressants prior to stroke.

The attrition rate for all participants was 34% (30% usual care, 41% intervention) at 6 weeks and 40% (36% usual care, 48% intervention) at 6 months. Further details of the follow-up data are given in the CONSORT diagram (Figure 3).

### 3.4. Objective D: Develop and Test Data Collection Systems, Outcome Measures and Follow-Up Protocols

#### 3.4.1. Data Collection Systems: Questionnaire Type

The number of completed questionnaires (270 at baseline, 156 (58%) at 6 weeks and 125 (46%) at 6 months) were summarised by questionnaire type at baseline, 6 weeks and 6 months, and, correspondingly, were (i) 183 (68%), 115 (74%) and 101 (81%) for the patient-reported questionnaire; (ii) 10 (3.7%), 5 (3%) and 5 (4%) for the patient-reported aphasia-friendly questionnaire; and (iii) 77 (28.5%), 36 (23%) and 19 (15%) for the consultee-reported questionnaire. The number of completed carer questionnaires at baseline, 6 weeks and 6 months were 259 (95.9%), 112 (71.8%) and 87 (69.6%), respectively.

The response rates for individual questionnaire items at baseline varied from 77% (FAST) to 100% (psychological input and self-reported psychological difficulties). Low response rates for the FAST questionnaire corresponded to more complex questions to comprehend being consistently unanswered.

#### 3.4.2. Data Collection Systems: Outcome Measures

Table 2 gives the number of participants with anxiety or depression based on each of the measures used. Rates of psychological distress were generally higher in carer-reported measures compared to patient-reported measures. The only exception was for the Yale, where carers reported lower rates than participants. The DISCs showed the lowest proportion of participants with depression among all depression measures.

At each follow-up point, if the participants scored above a pre-determined threshold indicating that they may be experiencing psychological distress, their GP was notified (14% at 6 weeks and 17% at 6 months).

To determine the reliability of participants’ self-reporting of further stroke, TIA and other major health problems, comparisons were made with hospital records at each follow-up. The agreement between self-reporting and hospital records was better at 6 weeks than 6 months; however, there were few problems reported by participants and in hospital records overall, leading to high agreement. The agreement analysis is described in Supplementary Materials Table S3.

#### 3.4.3. Follow-Up Protocol

A total of 105/229 (46%) participants at 6 weeks and 107/198 (54%) participants at 6 months received a postal or telephone reminder to return the questionnaire. Of those who received at least one reminder, at 6 weeks, 43 (41%) returned a questionnaire; at 6 months, 42 (39%) returned a questionnaire.

The overall response rate was low at both 6 weeks and 6 months. We conducted an analysis of non-responders to identify factors associated with non-response.

At 6 weeks, the following factors were statistically significantly associated with non-response to the follow-up questionnaire: participant age (with an increase in age, the odds of non-response were lower (95% CI 0.94 to 0.99)), questionnaire type (participants who completed the consultee version of the questionnaire had a higher probability of non-responding (95% CI 1.2 to 8.2)) and cognitive problems identified at baseline (a higher probability of non-responding (95% CI 1.1 to 5.8)).

Most participants who had not responded to the 6-week follow-up did not respond at 6 months (95% CI 11.1 to 79.9). At 6 months, participants with a consultee-completed questionnaire were more likely to not respond (95% CI 1.7 to 14.4). Other factors associated with non-response were receiving psychological support at baseline (95% CI 1.5 to 19.2), and living in supported accommodation, a care/nursing home or with relatives, compared to those living in their own home (95% CI 1.6 to 21.8). Participants who were unable to work or were retired were more likely to respond compared to those in full time employment (non-response 95% CI 0.01 to 0.9, and 95% CI 0.04 to 0.4, correspondingly). Most numbers in the analysis were low, which was reflected in the wide CIs.

Table 3 and Table 4 show the outcome data at 6 weeks and 6 months, respectively. The usual care and intervention groups scored similarly at both time points.

### 3.5. Objective E: Estimate the Proportion of People with Psychological Distress, Time to First Referral and Time to Treatment

#### 3.5.1. Estimating the Proportion of People with Psychological Distress

At baseline and at each follow-up point, most participants were able to have their psychological distress status classified using an algorithm (Appendix A). The proportion of participants that were unable to be classified by the algorithm due to missing data at each time point is given in Table 5.

Nearly half of all participants reported some form of psychological distress at baseline. There was an imbalance in the percentage of participants experiencing psychological distress at baseline, with the usual care group having more cases of anxiety, depression or either of the two (Table 5). The intervention and control groups were similar in terms of the level of psychological distress reported at 6 weeks. At 6 weeks, almost half (42%) had some form of psychological distress.

At 6 months, the proportion of participants with anxiety was lower, but those with depression was higher compared to at baseline and at 6 weeks. At 6 months, all 23 participants with anxiety also had depression (baseline 86%; 6 weeks 89%; 6 months 100%).

#### 3.5.2. Time to First Referral for Psychological Support/Treatment and First Treatment for Psychological Distress

Four participants were referred for psychological treatment during the study period. These participants received support 3 and 9 days after first being referred, respectively. The dates for the remaining two participants were not available.

Of those with psychological distress at baseline (*n* = 74), 28 (37%) had no treatment; of those with psychological distress first reported at 6 weeks and 6 months, 9/13 (69%) and 13/15 (87%) had no treatment. The percentages were similar for both the intervention and control groups.

### 3.6. Objective F: Explore the Potential Benefits of the Intervention Package for Patients, Including for Different Stroke and Socio-Economic Subgroups

Table 5 shows that in the usual care and the intervention group, anxiety was correspondingly 25% and 21% at 6 weeks, and at 6 months, in the usual care and the intervention group, depression was 48% and 40% and psychological distress was 48% and 40%. In the intervention group, the odds of anxiety at 6 weeks (OR = 0.74) and of depression (OR = 0.75) or either (OR = 0.72) at 6 months were lower compared to the controls (see Table 5). The trial was not powered to produce definitive results.

In addition, to explore the potential benefit of the IP for patients and assess the ‘promise’ of the intervention on psychological distress outcomes at 6 weeks and 6 months, the raw PHQ-9 and GAD-7 scores were analysed with a mixed-effects linear model. There was very little difference between the groups, with mean difference between the usual care and the intervention group in terms of the PHQ-9 at 6 weeks of 0.9 95%CI = (−1.1, 2.9) and at 6 months of 0.8 95%CI = (−1.3, 3.0); for GAD-7, the mean difference correspondingly at 6 weeks was 0.7 95%CI = (−1.3, 2.6) and at 6 months was −0.2 95% CI = (−2.0, 1.7), all adjusted for the baseline scores.

#### Potential Benefit of IPs for Patients and Subgroup Analysis of Socio-Economic Factors

**Figure 5:**
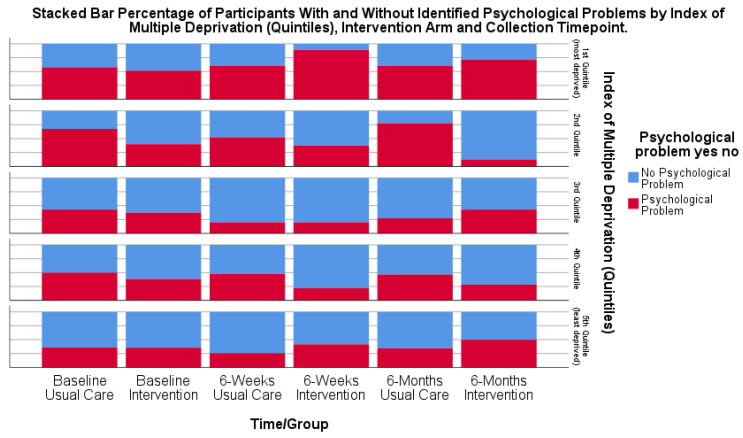
Bar chart of participants (%) with psychological distress at each time point by IMD quintile and intervention group.

The subgroup analyses of socio-economic factors (IMD) to explore the potential benefits for different socio-economic groups suggested that participants with higher deprivation (lowest quintile) were more likely to experience psychological distress post-stroke; however, the number of participants in each quintile was small. The proportion of participants with psychological distress was likely to remain at a similar level over time across all quintiles and for both the usual care and intervention groups (Figure 5).

Post-implementation process evaluation interviews with patients and carers following a stroke allowed for further exploration of the effectiveness of the IPs. Some patients described the psychological benefits of (i) staff being available and willing to initiate conversations about emotional changes following stroke and normalising the experience; (ii) being provided with information about the journey ahead to know what to expect; and (iii) access to ongoing support, including peer support and having the contact details of people who might provide support.

### 3.7. Objective G: Investigate the Feasibility of the Stepped-Wedge Design to Evaluate the Delivery of the Intervention Package

One significant challenge in the use of a stepped-wedge design was in implementing the IPs within the given timeframe. The study design initially comprised four time periods. However, during the ‘roll-out’ periods of the study, it became clear that the sites would not have enough time to agree to and complete the implementation of the IPs within the pre-specified timeframe. This included aspects of the IPs such as finalising key contact cards, patient information leaflets and setting up cross-service supervision links and staff training. It was therefore felt that an extension to the study was required, creating an additional fifth period of data collection (Figure 2) to allow extra time to ensure the IPs were agreed to and implemented, and that data collected during this fifth period would reflect a true ‘intervention’ period. However, despite the addition of a fifth period of data collection, the IPs were only partially implemented across the sites.

In the analysis, the measurements collected during the transition period were assigned as corresponding to the control group. We tested the sensitivity of the estimates of the intervention effect to this assumption and repeated the analysis for the dataset where participants recruited during the implementation period were assumed to have received the intervention. The sensitivity analysis results did not substantially differ from the main analysis (Supplementary Materials Table S4).

## 4. Discussion

This is the first study exploring the development and implementation of an intervention package for collaborative post-stroke psychological care within the NHS, social care and voluntary sector services. Whilst it seemed feasible to develop and implement the intervention package, implementing and evaluating this within a stepped-wedge RCT was challenging. The time taken to develop this complex intervention impacted its readiness to start being implemented. The data collection procedures/systems used may be feasible for a future study with the alterations proposed. Addressing these issues is essential for optimising future implementation and evaluation.

### 4.1. Feasibility of Stepped-Wedge Design

A multi-site stepped-wedge RCT requires the sites to be ready for randomisation and intervention implementation at the same pre-determined time. Therefore, our sites had to achieve collaborative intervention package development (pathway, training, manual, supervision) and to implement it into practice within three months; however, this took longer than three months. Future studies should consider the following:

- A longer pre-implementation preparation period: Allocating a longer dedicated preparation phase (e.g., 6–9 months) prior to implementation may ensure readiness.
- Implementation support teams: Establishing local implementation leads or teams within each site could facilitate adaptation to service structures and improve engagement.

Achieving staff buy-in and successful implementation requires the early engagement [48,49] of a range of staff. The development of the intervention package necessitated collaboration from the start by multiple professions and lay representatives from a range of services and organisations across each site’s care pathway. These services had mostly not previously collaborated, yet it was necessary here, as the intervention traversed service boundaries. However, stakeholder meetings were well attended, and staff felt the collaboration between stroke and NHS Talking Therapies services was beneficial. We did experience the known complexities of gathering stakeholders for collaborative meetings [50]; some key staff, e.g., clinical psychologists, did not attend. Furthermore, as the intervention required wide changes to implement all four components, there were delays, and all sites struggled to develop the intervention within the timeframe. Practical strategies to enhance engagement include the following:

- Incentives and recognition: Providing professional development credits or recognition for engagement in implementation efforts.
- Tailored communication strategies: Regular, targeted communication (e.g., newsletters, briefing sessions) to maintain momentum.

Previous stepped-wedge design studies of complex interventions have predominantly evaluated single component interventions, or a previously developed complex intervention, so these interventions were implementation-ready. In our study, we involved site staff in developing the intervention packages and in tailoring them to the different site service configurations. The intervention packages’ pathways and manual components needed to be authorised by senior leaders pre-implementation, which one site failed to achieve. Implementation may be more successful with early leadership buy-in, which then guides service delivery staff [50]. While we largely achieved early leadership buy-in, one site’s senior leader did not sign-off the intervention package. This may have impeded this site’s staff engagement and ownership, as well as the package’s full implementation. To address this in future studies, the following are recommended:

- Alignment strategies with service development: Aligning intervention components with existing service priorities may enhance acceptability and integration.
- Clear accountability: Defining responsibilities in implementation plans to ensure engagement.

Patient and carer involvement in intervention package development was important and led to the creation of patient-facing materials. This generated additional complexities as these materials required approval from different committees (e.g., Patient Involvement Groups) before being implemented. These groups did not meet regularly, or had a backlog of documents to review, creating further delays, and materials were not approved in all sites.

Delivering staff training took longer than the intended three months. Whilst staff were keen to receive training and it was well received, releasing staff to attend training was challenging. In stroke services, there was a greater representation of allied health professionals compared to nursing staff due to the nursing workload. In one site, training was embedded into standard service training. A future study may consider a similar approach. In one site, the inpatient psychology service agreed to deliver training to NHS Talking Therapies. However, despite frequent reminders, this was not delivered within the timeframe. We tried to be flexible in training delivery by allowing shorter sessions and using a train-the-trainer approach. Despite this, training went beyond the three-month implementation phase, with some sites training staff up to eight months later. To enhance training efficiency, future studies could consider the following:

- Flexible training delivery: Offering asynchronous online training modules with optional live question and answer sessions which may improve accessibility.
- Integrating training into service training programmes: Embedding training into existing professional development frameworks which may reduce disruption.

It was feasible to implement supervision, although it took different forms across sites. Supervision and mutual support between NHS Talking Therapies and stroke services were more successful in sites with pre-existing relations between teams. In sites without these pre-existing links, supervision was agreed, and connections developed, but these were less embedded by the study close. Mutual support and willingness to seek support may have been facilitated by established relationships. A longer implementation phase may have allowed relationships to develop between services and increased the likelihood of successful implementation. This is especially important in stroke services without access to clinical psychology, where guidance from NHS Talking Therapies would facilitate stroke staff in providing psychological support.

This pragmatic stepped-wedge cluster randomised trial is particularly well suited for heterogeneous clusters with a substantial cluster-level effect when there is evidence to support the intervention, or where sites may not wish to be randomised into the control arm [27]. However, there is the possibility that some clusters will be unable to initiate the intervention according to the pre-specified schedule, as happened in our study. As implementation exceeded the planned three-month period, we attempted to ensure that the intervention phase was truly reflected by adding a data collection period to the end of the study. Given the difficulties encountered in a four-site stepped-wedge trial, involving a greater number of sites in such a study would be challenging. A future study should consider an alternative design, e.g., a straightforward cluster randomised trial, where the implementation period is less time-bound.

### 4.2. Feasibility of Data Collection Procedures/Systems

Our data collection procedures were somewhat feasible but may require alterations for a future study. All sites successfully achieved recruitment and baseline data collection. Our eligibility rate was 63%, and 40% of those eligible were recruited. These figures are lower than expected based on other studies of complex interventions [51]. We widened inclusion, including those with aphasia, cognitive impairment and consultee assent. However, for some patients, consultees were not available, and overall, 37% were ineligible. Additionally, one-third of those eligible were discharged from hospital before being approached about the study.

Follow-up data collection was affected by an attrition rate of 34% at 6 weeks and 40% at 6 months; this high attrition may be in part because patients were recruited early after stroke, although this rate is lower than in another study exploring the collaborative delivery of psychological interventions [52]. The higher attrition among participants with consultees (<50% of these participants returned 6-week data, 25% at 6 months) suggests that a future study should reconsider the use of consultees given the attrition in these participants. Overall, data completion in the returned questionnaires was high. However, despite PCPI engagement to ensure the suitability of the questionnaires, completion was lower for some measures. The Work and Social Adjustment Scale (WSAS) had the highest non-completion rate. This scale was included as it is routinely collected in NHS Talking Therapies, and a stepped-wedge design is more suited to routinely collected data, as the control and intervention phases both become usual care. Within this, there was one question about work, which was not applicable for many participants. This question may not be suitable for use in post-stroke studies, and therefore its inclusion should be questioned for future studies, where an alternative design may be used, following discussion with and input from a PCPI group. Strategies to improve recruitment and retention might include the following:

- Post-discharge recruitment pathways: Allowing recruitment after hospital discharge.
- Personalised follow-up strategies: Using reminder calls, SMS messages and flexible follow-up options (e.g., visits, virtual check-ins) may improve retention.
- Simplified data collection: Streamlining questionnaires to reduce participant burden may improve response rates.

Participants’ self-reporting of subsequent health problems and resource-use was fairly accurate when compared with electronic hospital records. However, with only small numbers of health problems recorded overall, interpreting the accuracy of the self-reported information was difficult. It may be feasible for a future definitive study to use electronic health records, reducing the burden on participants, whilst still obtaining relevant, accurate data for economic evaluation.

Our study-specific algorithm for classifying participants as being in psychological distress or not allowed for a high proportion (96.8–100%) of participants to be classified, depending on psychological distress type and proxy completion. However, carer-completed questionnaires were not efficient in identifying psychological distress, and could therefore be removed from a future study’s algorithm.

Overall, a high number of participants had psychological distress, which reflects the wider literature [53,54]. Despite this, very few participants were referred for or received support. However, our collection of these data had limitations. Firstly, referral data were only collected from hospital records, and referrals made elsewhere (e.g., GPs) may have been missed. Secondly, follow-up questionnaires did not ask about referrals. It is possible that some participants had been referred, but were not seen by the follow-up time, as our scoping exercise showed extensive waiting list times, e.g., up to nine months for NHS Talking Therapies. Thirdly, we did not capture the informal support participants may have received, such as supportive conversations with stroke staff, which was the focus of our training. A future study should include the collection of referral data from multiple sources, informal support received and the standard of psychological care received, to determine intervention effectiveness.

Participant outcomes of psychological distress appeared to be related to socio-economic status. Although only small numbers of participants represented each deprivation index quintile, there was a trend towards participants living in higher deprivation areas being more likely to experience psychological distress. While only suggestive, this trend is reflective of evidence from the general population, highlighting the link between area deprivation and mental health [55]. Psychological support may be limited, or there may be more challenges to accessing services. Psychological services and interventions should therefore incorporate strategies to ensure equitable access. These might include the following:

- Targeted outreach: Proactive engagement in underserved areas to improve accessibility.
- Flexible service delivery models: Offering telephone or virtual psychological support to help to reduce barriers.

Whilst this study has provided valuable insights into developing and implementing a collaborative post-stroke psychological care model, this study did have some limitations. Firstly, the intervention was developed and tested within specific NHS and social care contexts, so its findings may not fully translate to other healthcare systems or resource-limited settings. Secondly, as staff were actively involved in developing the intervention, there may have been a positive bias in their perception of its feasibility and acceptability. Lastly, the study relied partly on self-reported psychological distress and service use, which may have introduced recall bias or underreporting. Despite these limitations, the study provided crucial insights into the feasibility of integrating psychological care into post-stroke services and highlighted key areas for future improvement.

## 5. Conclusions

In conclusion, it was feasible to collaboratively develop intervention packages and tailor all four components in all sites. Implementation was feasible for most sites but was affected by timeframes associated with the stepped-wedge design and service processes. Intervention packages, when implemented, were generally well received by staff who noticed an overall increased focus on psychological support. The stepped-wedge design meant that all sites received the intervention and, having wanted to increase post-stroke psychological provision, were all able to participate in influencing this. However, an alternative study design should be considered for a future study to facilitate the implementation of this complex intervention, with adapted data collection procedures to evaluate effectiveness. Practical recommendations include a longer implementation period, alternative trial designs, and targeted strategies for improving access and retention. Future research should explore the long-term impact of collaborative psychological interventions, assess cost-effectiveness and refine implementation strategies to optimise their scalability within routine NHS and social care settings.

## Figures and Tables

**Table 1:** Baseline participant characteristics, demographics and stroke and mood measures by intervention group.

	Usual Care (*N* = 179)	Intervention (*N* = 91)	All (*N* = 270)
Age, median (IQR) *n* = 269	72 (62, 81)	76 (61, 83)	73 (62, 82)
Gender ^, *n* (%)			
Female	85 (47.8)	44 (48.4)	129 (48.0)
Ethnicity ^^, *n* (%)			
White	172 (97.2)	87 (95.6)	259 (96.6)
Employment status ^^, *n* (%)			
Paid	37 (20.9)	16 (17.6)	53 (19.8)
Living situation ^^, *n* (%)			
At Home	149 (84.2)	78 (85.7)	227 (84.7)
Index of Multiple Deprivation (quintiles), *n* (%)			
1st (most deprived)	45 (25.1)	32 (35.2)	77 (28.5)
2nd	37 (20.7)	16 (17.6)	53 (19.6)
3rd	21 (11.7)	16 (17.6)	37 (13.7)
4th	38 (21.2)	13 (14.3)	51 (18.9)
5th (least deprived)	38 (21.2)	14 (15.4)	52 (19.3)
Type of stroke ^^^, *n* (%)			
Ischaemic	145 (81.9)	86 (95.6)	231 (86.5)
Intra-Cerebral Haemorrhage	32 (18.1)	4 (4.4)	36 (13.5)
Side of body affected by stroke ^^^, *n* (%)			
Left	76 (43.2)	38 (41.8)	114 (42.7)
Right	83 (47.2)	43 (47.3)	126 (47.2)
Bilateral	2 (1.1)	2 (2.2)	4 (1.5)
Neither	15 (8.5)	8 (8.8)	23 (8.6)
NIHSS score, median (IQR) *n* = 210	4 (2.5, 8.5)	5 (2, 11)	5 (2, 10)
Estimated Barthel Index, median (IQR) *n* = 265	16.3 (10, 20)	17.5 (10, 20)	17.5 (10, 20)
Modified Rankin ^^, *n* (%)			
Moderate to Severe	89 (49.7)	42 (46.2)	131 (48.9)
EQ5—VAS, median (IQR) *n* = 188	55 (40, 75)	70(50, 80)	60 (50, 80)
Sensory impairment (sight or hearing), *n* (%)	61 (34.1)	31 (34.1)	92 (34.1)
Cognitive score (MOCA), median (IQR) *, *n* = 181	23 (18, 26)	24 (19, 27)	23 (18, 26)
Cognitive impairment, n (%) *, *n* = 181	90 (73.8)	37 (62.7)	127 (70.2)
Communication score (FAST), median (IQR) *, *n* = 148	29 (26, 30)	29 (25, 30)	29 (26, 30)
Communication problems, n (%) *, *n* = 148	19 (18.6)	11 (23.9)	30 (20.3)
Current/past use of anti-depressants ^^, *n* (%)	36 (20.3)	7 (7.7)	43 (16.0)
Current/past use of psychological support ^^^, *n* (%)	31 (17.6)	10 (11.0)	41 (15.4)
Self-reported psychological difficulties, *n* (%)	80 (44.7)	39 (42.9)	119 (44.1)

* Not applicable to participants with consultee form completion. Missing data: ^ *n* = 1; ^^ *n* = 2 and ^^^ *n* = 3.

**Table 2:** Number of participants (%) deemed to have anxiety or depression from each questionnaire at baseline by intervention group.

Questionnaire	Problem *^1^	Usual Care *n* (%)	Intervention *n* (%)	All Participants *n* (%)
GAD-7 *^2^	Anxiety	26 (21.3)	11 (18.0)	37 (20.2)
BOA	Anxiety	50 (29.4)	22 (25.9)	72 (28.2)
PHQ-9 *^2^	Depression	29 (24.4)	10 (16.4)	39 (21.7)
SADQ-10	Depression	52 (30.1)	21 (24.4)	73 (28.5)
Yale *^3^	Depression	42 (33.6)	23 (35.9)	65 (34.4)
Carer Yale	Depression	52 (30.6)	20 (23.3)	72 (28.1)
DISCs	Depression	22 (17.6)	10 (15.6)	32 (16.9)

*^1^ Problem is determined by dichotomising the total score from each questionnaire. *^2^ Only applicable to returned participant-completed questionnaires. *^3^ Not applicable to returned consultee-completed questionnaires.

**Table 3:** Six-week participant measures by intervention group.

	Usual Care (*n* = 108)	Intervention (*n* = 48)	All (*n* = 156)
Estimated Barthel Index, median (IQR) *n* = 155	20 (16.3, 20)	20 (15, 20)	20 (15, 20)
Modified Rankin, *n*(%)			
Moderate to severe	48 (44.4)	22 (47.8)	70 (45.5)
EQ5—VAS, median (IQR) *n* = 119	70 (50, 87)	70 (55, 90)	70 (50, 90)
SF—SIS, median (IQR) *n* = 99	31 (23, 37)	30 (20, 37)	30.5 (23, 37)
WSAS, median (IQR) *n* = 123	12 (4, 28)	10.5 (2, 26)	12 (2, 28)
IES-6, median (IQR) *n* = 143	1 (0.5, 2)	1 (0.2, 2.2)	1 (0.3, 2)

**Table 4:** Six-month participant measures by intervention group.

	Usual Care (*n* = 87)	Intervention (*n* = 38)	All (*n* = 125)
Estimated Barthel Index, median (IQR) *n* = 122	20 (17.5, 20)	20 (15, 20)	20 (17.5, 20)
Modified Rankin, *n*(%)			
Moderate to severe	35 (40.2)	15 (39.5)	50 (40.0)
EQ5—VAS, median (IQR) *n* = 103	70 (50, 80)	70 (65, 90)	70 (50, 85)
SF—SIS, median (IQR) *n* = 115	32 (24, 37)	30 (21, 38)	32 (22, 37)
WSAS, median (IQR) *n* = 103	8 (1, 25.5)	7 (0, 28)	8 (0, 26)
IES-6, median (IQR) *n* = 114	0.8 (0.3, 1.7)	0.8 (0.3, 2)	0.8 (0.3, 1.8)

**Table 5:** Number of participants in psychological distress (%) and estimates of intervention effects by time point for anxiety, depression or either.

	Usual Care	Intervention	Total	Missing	Adjusted OR * (95% CI)
**Baseline**	***n*** **= 179**	***n*** **= 91**	***n*** **= 270**		
Anxiety	52 (29.1)	22 (24.2)	74 (27.4)	5 (1.9)	N/A
Depression	84 (46.9)	36 (39.6)	120 (44.4)	2 (0.7)	N/A
Either	92 (51.4)	38 (41.8)	130 (48.2)	4 (1.5)	N/A
**6 Weeks**	***n*** **= 108**	***n*** **= 48**	***n*** **= 156**		
Anxiety	27 (25.0)	10 (20.8)	37 (23.7)	4 (2.6)	0.74 (0.28, 1.93)
Depression	42 (38.9)	19 (39.6)	61 (39.1)	0 (0.0)	1.18 (0.55, 2.50)
Either	45 (41.7)	20 (41.7)	65 (41.7)	1 (0.6)	1.06 (0.50, 2.26)
6 Months **	***n*** **= 87**	***n*** **= 38**	***n*** **= 125**		
**Anxiety**	16 (18.4)	7 (18.4)	23 (18.4)	4 (3.2)	1.02 (0.35, 2.98)
**Depression**	42 (48.3)	15 (39.5)	57 (45.6)	0 (0.0)	0.75 (0.31, 1.79)
**Either**	42 (48.3)	15 (39.5)	57 (45.6)	3 (2.4)	0.72 (0.30, 1.77)

* Adjusted for corresponding psychological distress status at baseline. ** Potential contamination for roll-out period included in model.

## Data Availability

The original contributions presented in this study are included in the article/Supplementary Materials. Further inquiries can be directed to the corresponding author.

## Supplementary Materials

The following supporting information can be downloaded at: https://www.mdpi.com/article/10.3390/healthcare13070824/s1, Supplementary Table S1: Baseline study measures; Supplementary Table S2: The 6-week/6-month study measures; Supplementary Table S3: Hospital admissions—number (%) of participants with further stroke, TIA or other major health problems if electronic and patient-completed forms are available; Supplementary Table S4: Number of participants in psychological distress (%) and estimates of intervention effects at 6 weeks and 6 months for anxiety, depression or either, assuming those recruited in the roll-out period received the intervention.

## Appendix A

### Psychological Distress Algorithm

**Psychological distress due to anxiety**

(1) A patient will be identified as being in psychological distress for anxiety using the following algorithm:
  (i) If a patient does not have a consultee and does not have aphasia, they will be identified as being in psychological distress due to anxiety if they scored 10 or more on the GAD-7.
  (ii) If the patient has not completed the patient self-completed questionnaire and does have a consultee or carer, then the corresponding measure (GAD-7 for consultee and BOA for carer questionnaire) will be used to indicate whether a patient is in psychological distress due to anxiety. If, for either of the corresponding measures, the patient has scored above the cut-off (10 or more on the GAD-7 and 14 or more on the BOA), then the patient be identified as being in psychological distress due to anxiety.
(2) If the patient is identified as not being in psychological distress due to anxiety from either the GAD-7 or BOA, and is not identified as being in psychological distress due to anxiety from (1) above, then they will be identified as not being in psychological distress due to anxiety.
(3) If, from (1) and (2) above, the patient cannot be classed as either being in psychological distress due to anxiety or as not being in psychological distress due to anxiety, their status for psychological distress due to anxiety will be set to ‘missing’.
(4) If their status for psychological distress due to anxiety is missing and the unused measure from (1) above indicated psychological distress, then they will be indicated as being in psychological distress due to anxiety.

**Psychological distress due to depression**

(1) A patient will be identified as being in psychological distress due to depression using the following algorithm:
  (i) If a patient completes the patient self-completed questionnaire (that is, they do not have a consultee and do not have aphasia), they will be identified as being in psychological distress due to depression if they scored 10 or more on the PHQ-9.
  (ii) If a patient does not have a consultee but does have aphasia, they will be identified as being in psychological distress due to depression if they scored 3 or more on the DISCs.
  (iii) If a patient has responded ‘Yes’ to the Yale question (on either the patient self-completed or aphasia-friendly patient questionnaire).
  (iv) If the patient has not completed the patient self-completed questionnaire and does have a consultee or carer, the consultee and/or carer questionnaire will be used to indicate whether a patient is in psychological distress due to depression. If a carer scored their relative/friend as 14 or more on the SADQ-10, then the patient would be identified as being in psychological distress due to depression; likewise, if the relative/friend is screened positive for mood on the Yale question on either the consultee or carer questionnaire, the patient will be identified as being in psychological distress due to depression.
  (v) A patient will also be identified as being in psychological distress due to depression if a letter has been sent to their GP.
(2) If the patient is identified as not being in psychological distress due to depression from any of the measures detailed in (1) (i)–(iv) above (PHQ-9; DISCs; patient, consultee or carer Yale question) and is not identified as being in psychological distress due to depression in (1) (v) above, then they will be identified as not being in psychological distress due to depression.
(3) If, from (1) and (2) above, the patient cannot be classed as either being in psychological distress due to depression or as not being in psychological distress due to depression, their status for psychological distress due to depression will be set to ‘missing’.
(4) If their status for psychological distress due to depression is missing and an unused measure from (1) above indicated psychological distress, then they will be indicated as being in psychological distress due to depression.

**Psychological distress variable**

(1) A patient will be identified as being in psychological distress if they are recorded as having psychological distress due to anxiety or recorded as having psychological distress due to depression (or both).
(2) A patient will be identified as not being in psychological distress if they are recorded as not having psychological distress due to anxiety and recorded as not having psychological distress due to depression (or both).
(3) If a patient is not recorded as being in psychological distress and is recorded as ‘missing’ on either (or both) psychological distress due to anxiety and psychological distress due to depression, then they will be recorded as ‘missing’ for psychological distress.
