# Supplementary material for: Accelerating the Delivery of Psychological Therapies After Stroke: A Feasibility Stepped-Wedge Cluster Randomised Controlled Trial
Source: Healthcare (Basel). 2025 Apr 4;13(7):824. doi: 10.3390/healthcare13070824 (PMC11988650; doi:10.3390/healthcare13070824)
Supplement: Supplementary file 1 [file healthcare-13-00824-s001.zip › healthcare-3525879-supplementary.pdf]

ADOPTS Supplementary Material

Supplementary Table S1. Baseline study measures

	Questionnaire			
	OR		*OR*	*PLUS*
	Patient self-complete	Aphasia-friendly patient	Consultee	Carer
**Demographics**	X	X	X	
**Social/economic characteristics**	X	X	X	
**Cognition**				
MoCA (30-items)	X	X		
**Communication**				
FAST (22-items)	X	X		
**Mood**				
PHQ-9 (9-items)	X			
Self-reported/diagnosis psychological difficulties since stroke	X	X	X	
Use of medication for psychological difficulties	X	X	X	
Psychological input including timings	X	X	X	
SADQ-10 (10-items)				X
**Aphasia-friendly mood scales**				
DISCs (1-item)	X	X		
Yale (1 item)	X	X	X	X
**Anxiety**				
GAD-7 (7-items)	X			
BOA (10-items)				X
**Stroke recovery**				
Barthel (3-items)	X	X	X	
Modified Rankin (1-item)	X	X	X	
**Quality of life**				
EQ5D3L (5-items)	X	X		
**Pre-existing sensory impairment**				
	X	X	X	

Supplementary Table S2. 6-week/6-month study measures

	Questionnaire			
	OR	*OR*	*PLUS*	
	Patient self-complete	Aphasia-friendly patient	Consultee	Carer
**Social/economic context**	X	X	X	
**Mood**				
Self-reported or diagnosis psychological difficulties since stroke	X	X	X	
Medication for psychological difficulties	X	X	X	
Psychological input including timings	X	X	X	
PHQ-9	X			
SADQ-10				X
**Aphasia-friendly mood scales**				
DISCS	X	X		
Yale single item	X	X	X	X
**Anxiety**				
GAD-7	X		X	
BOA				X
**PTSD**				
IES-6	X		X	
**Stroke recovery**				
Further stroke	X	X	X	
Barthel	X	X	X	
Modified Rankin	X	X	X	
Short Form Stroke Impact Scale	X	X	X	
**Quality of life**				
EQ5D3L	X	X		
**Participation**				
WSAS	X	X	X	

Supplementary Table S3: ***Hospital admissions – number (%) participants with further stroke, TIA or other major health problems if electronic and patient completed forms available.***

	Reported in health records N (%)	Self-reported by participant N (%)	Reported In Both N(%)	Kappa (95% CI)	Total Agreement N(%)
**6 weeks**					
Stroke	2 (2.0)	3 (2.9)	1 (1.0)	0.39 (-0.17 to 0.94)	99 (97.1)
TIA	2 (2.0)	4 (3.9)	1 (1.0)	0.32 (-0.18 to 0.81)	98 (96.1)
Other problems	10 (9.8)	10 (9.8)	2 (2.0)	0.11 (-0.14 to 0.36)	86 (84.3)
Any	14 (13.7) N= 102	17 (16.7) N= 102	4 (3.9) N= 102	0.13 (-0.10 to 0.35)	79 (77.5)
**6 months**					
Stroke	0 (0.0)	1 (1.2)	0 (0.0)	0.29 (0.01 to 0.58)	81 (98.8)
TIA	1 (1.2)	0 (0.0)	0 (0.0)	N/A	81 (98.8)
Other problems	12 (14.6)	8 (9.8)	3 (3.7)	0.21 (-0.07 to 0.49)	68 (82.9)
Any	12 (14.6) N= 82	9 (11.0) N= 82	4 (4.9) N= 82	0.29 (0.01 to 0.58)	69 (84.1)

***Supplementary Table S4*: Number of participants in psychological distress (%) and estimates of intervention effect at 6-weeks and 6-months for anxiety, depression or either assuming those recruited in the roll-out period received the intervention.**

	Usual care	Intervention**	Total^	Adjusted OR* (95% CI)
**6-Weeks**	N = 59	N=97	N=156	
**Anxiety**	14 (24.1)	23 (24.5)	37 (24.3)	0.92 (0.40, 2.11)
**Depression**	23 (39.0)	38 (39.2)	61 (39.1)	1.01 (0.49, 2.07)
**Either**	24 (40.7)	41 (42.7)	65 (41.9)	1.15 (0.56, 2.37)
**6-Months****	N=42	N=83	N=125	
**Anxiety**	7 (16.7)	16 (20.3)	23 (19.0)	0.64 (0.14, 2.82)
**Depression**	21 (50.0)	36 (43.4)	57 (45.6)	0.72 (0.18, 2.90)
**Either**	21 (50.0)	36 (45.0)	57 (46.7)	0.90 (0.22, 3.66)

* Adjusted for corresponding psychological distress status at baseline.
** Potential contamination for rollout period included in model.
^ Sample sizes include missings, numbers for missing data are in Table 4
